# Automated Reinforcement Management System (ARMS): focused phase I provider feedback

**DOI:** 10.1186/s13722-022-00301-w

**Published:** 2022-03-26

**Authors:** Crystal L. Smith, Nicole M. Rodin, Julie Y. Hwang, André Q. C. Miguel, Kim Johnson, Michael G. McDonell, Sterling M. McPherson

**Affiliations:** 1 Elson S. Floyd College of Medicine, Washington State University, PO Box 1495, Spokane, WA 99210-1495 USA; 2 Analytics and PsychoPharmacology Laboratory (APPL), Spokane, WA USA; 3 Program of Excellence in Addiction Research (PEAR), Washington State University, Spokane, WA USA; 4 College of Pharmacy and Pharmaceutical Sciences, Washington State University, Spokane, WA USA; 5 Managed Health Connections, Spokane, WA USA; 6 Behavioral Health Innovations, Washington State University, Spokane, WA USA

**Keywords:** Digital therapeutic, Contingency management, Alcohol use disorder, Novel treatment

## Abstract

**Background:**

Alcohol use increases risk for morbidity and mortality and is associated with over 3 million annual deaths worldwide. Contingency Management (CM) is one of the most effective interventions for substance use disorders, and has recently been coupled with technologies to promote novel treatments for alcohol use disorders (AUD). Leveraging these technological advances, we are developing the Automated Reinforcement Management System (ARMS), an integrated CM system designed to enable CM treatment as a component of a digital therapeutic or adjunct therapy remotely to anyone with a smartphone.

**Objective:**

To collect detailed provider feedback on ARMS and determine the need for modifications to make the system most feasible, acceptable, and useful to providers.

**Methods:**

Seven providers completed one-hour structured interviews/focus groups wherein we described the ARMS system and its application to clinical care. Providers viewed screen shots of the ARMS provider facing and patient facing systems. Providers gave feedback on their current AUD treatment practices, preferences for the functionality and appearance of the system, preferences for receipt of information on their patients, why they and their patients would or would not use the system, suggestions for improvement, and the proposed intervention overall. To analyze the qualitative data gathered, we used a qualitative descriptive approach with content analysis methods.

**Results:**

The overarching theme of *Individualized Treatment* emerged throughout the interviews. This sentiment supports use of ARMS, as it is intended to supplement provider communication and intervention as an adjunctive and customizable tool with the ability to reach rural patients, not a stand-alone option. Themes of *Accountability* and *Objective Assessment* arose during discussions of why people would use the system. Themes within provider obstacles included*, Information Overload* and *Clinical Relevance*, and in patient obstacles, *Sustained Engagement* and *Security Concerns*. Two themes emerged regarding suggestions for improvement: *Increasing Accessibility* and *Bi-directional Communication*.

**Discussion:**

Themes from provider input are being used to modify ARMS to make it more user friendly, time saving, and relevant to treatment of AUD. If successful, ARMS will provide effective, individualized-digital therapeutic for those needing adjunctive treatment or those living in rural remote areas needing better connected care.

## Introduction

Alcohol use increases risk for morbidity and mortality and is connected to over 3 million annual deaths worldwide [1]. It has been identified as a causal factor in over 200 conditions, including infectious diseases, nutritional conditions, cancers, and injuries [1, 2]. In the United States, comparison of rates of alcohol use between 2001–2002 and 2012–2013 indicate an increase in DSM-IV Alcohol Use Disorder (AUD) of nearly 50%, from 8.5 to 12.7% [3].

With a long history of effective application for many substance use disorders [4–6], Contingency Management (CM) has been identified as a cost-effective behavioral treatment approach and has more recently increased its applicability for AUDs in light of recent technological advancements [7]. The basic premise of CM for substance use consists of providing reinforcement (i.e., incentives such as financial rewards, gift cards, toiletries, or electronics) following objective verification of substance abstinence, usually through a biospecimen [8–10]. Although the efficacy of CM for many substance use disorders is well documented [4–6], only a small number of studies have evaluated the efficacy of CM for AUD [11]. This is primarily due to the lack of biomorakers able to detect longer periods of alcohol consumption as, until recently, the only commercially available alcohol use biomarker was breath alcohol content (BAC) that, while acurate, can only detect alcohol use up to 12 h after consumption.

The increased availability and widespread use of mobile systems provides a new and innovative arena for the treatment of AUD using this technology, particularly for rural patients who may have more difficult access to care. Recent advances have provided the opportunity to use mobile systems in conjunction with low-cost consumer electronic breathalyzer devices to monitor alcohol use remotely [12]. The few studies that have examined this combination of technology and CM for AUD have been successful in supporting participants in achieving alcohol abstinence [13–15]. There remains an important translational gap in this area of research, however and even though CM has recently been included in California’s Medi-Cal Policy to be used in conjunction with multiple evidence-based modalities, studies have not examined whether or how this therapeutic combination will work in real-life community treatment settings.

Qualitative research conducted with focus groups have been used in software system development and translational clinical research to efficiently provide a broad perspective from various participants. This has been documented more within the public health realm, but is highly relatable to the concept of using diverse provider perspective to drive system development and clinical implementation [16–18]. By gathering providers from various practice sites, participants have shown benefit in stimulating ideas that generate both breadth and depth on the topic to further advance perspectives on the subject. These diverse perspectives have been used within evidence-based literature to identify preliminary data and shape potential research questions for clinical decision making [16].

The Automated Reinforcement Management System (ARMS) is a hybrid mobile/internet operating system (IOS) Web CM system designed by Managed Health Connections to integrate with treatment programs, providing clinicians with an adjunctive treatment modality to: (1) administer CM treatment for patients with AUD remotely; (2) access updated patient information that can be critical to the clinical management of AUD; and (3) communicate safely and directly with patients through the ARMS mobile component. All data collected from patients using the mobile component are stored securely in a database that can be accessed by providers using their Provider Dashboard. On this dashboard providers can register new patients, customize alerts they can receive based on patient factors such as non-adherence, view all patient breath results, Ecological Momentary Assessment (EMA) submissions and see patients who are highlighted as potentially struggling. A critical aspect of the CM component involves providing rewards contingent on the objective verification of alcohol abstinence. The ARMS system is designed to provide electronic gift cards (e.g., to Amazon or Walmart) that can also be sent to the patient’s email and/or printed [19]. Patients are prompted by the mobile component to provide a breath sample, while using facial recognition on their mobile device to ensure the correct person is submitting the sample. The patient mobile interface syncs via Bluetooth with a breathalyzer device, enabling the recording and submission of the breathalyzer sample. The application can send the patient messages confirming that their sample has been submitted and summarizing the rewards they have earned.

The objective of this study was to collect detailed provider feedback on ARMS and determine the need for future modifications that would render the system most feasible, acceptable, and useful to providers, thus maximizing its chances for implementation in real treatment settings.

## Methods

### Participants

Clinical providers, based in Idaho and Washington State, were recruited to participate in one-hour long semi-structured interviews or focus groups (depending on their availability) via the Zoom video conferencing platform. Requirements for participation included being 18 years of age or older, speaking English, working as a clinical provider with people who have AUD or are heavy drinkers and having the ability to provide e-consent.

### Study design

One-hour interviews/focus groups wherein researcher CLS described the ARMS system and its application to clinical care were conducted. Providers viewed screen shots of the ARMS provider facing and patient facing systems. Providers gave feedback on each of the following items, which were explicitly asked in open ended questions: current practices for tracking progress in AUD treatment including frequency in measuring progress, frequency of communication with patients and primary contact person for communication, their preferences for the functionality and look of the application, which data they would prefer to have highlighted at a glance (e.g., number of hot zone visits where patients are in locations that may trigger alcohol use, percent of positive BAC readings, number of BAC requests fulfilled by the participant, highest BAC reading for the patient, number of responses sent or received to ecological momentary assessment questions, rewards earned, etc.). Providers were also asked what they would consider to be indicators of risk that they would like to follow up with patients on (e.g., failure to fulfill a BAC or EMA request or having a high craving level), how and how often they would prefer to receive information on their patients, why they and their patients would or would not use the system, potential obstacles that may be encountered with the use of this system, their suggestions for improvement, and the proposed intervention in general. Focus groups were recorded and transcription was conducted by a paid, professional transcriptionist under a confidentiality agreement. For a full description of the ARMS project protocol see Miguel et al. [19].

### Analysis

To evaluate the qualitative data provided by the focus groups and interviews we elected to use a qualitative descriptive approach [20, 21] with content analysis methods based on recommendations by Schreier [22]. This approach allowed us to identify common themes amongst the provider feedback. Open ended questions were asked of participants to gather information on the following areas: current practices, preferences for the application, thoughts about why people would or would not use the application, and suggestions for improvement.

Following transcription, qualitative analysis was conducted using the following steps: transcripts were read in full and marginal notes (e.g. analyst reflections, reactions, queries) were made by researchers CLS and NMR. Both qualitative analysts established preliminary codes and themes that appeared within the transcripts. The analysts met to discuss their individual preliminary efforts and discuss consistency and inconsistency amongst their codes and themes. All inconsistencies were discussed until the analysts arrived at agreement. Both analysts then coded the transcripts, assigning individual sections of data summative, truncated codes that captured the salient components of the data, assisting with progressive organization and interpretation. The codes and themes identified were then examined in a process of interrelation; an examination of the relationship between, within and among them [23]. Transcripts were then re-read, and the data evaluated for its fit into the themes in an iterative process. Themes were then evaluated for overlap and adjusted so they were unique and exclusive. To address reliability, an audit trail was kept by the primary analyst throughout the analysis process to document decisions and progress.

## Results

Seven clinical providers (four female) participated in the interviews. Years of experience with patients who have AUD ranged from 6 to 39 (*m* = 16.57*, sd* = 11.31) and approximate percentage of patients serviced in their practice with AUD ranged from 4 to 50% (*m* = 14.86*, sd* = 16.18). The current professions that participants held varied from physicians (primary care to emergency department), to academics, therapists, and a recovery support navigator. Provider ages ranged from 37 to 65 (*m* = 49.86*, sd* = 9.69). All but one provider self-identified as non-Hispanic White and one self-identified as having both ethnicity and race of Hispanic/Latino.

Both analysts agreed on coding and theme development with 100% continuity at the end of the iterative qualitative analysis process, wherein the analysts met three times to establish and discuss coding and theme development. Separate themes were identified for each content area; however, a pervasive overarching theme was found throughout the interviews; *Individualized Treatment*. Providers emphasized that recovery is an individualized process and while enthusiastic about the potential of technology-based interventions, they explained that this intervention would best serve patients and providers if it was flexible and adjunctive to treatment by trained providers.

When asked about their current practices, providers explained that the practices were *Setting and Patient Progress Dependent.* Providers explained that progress tracking, frequency and method of communication with patients were dependent on the patient’s progress in therapy, describing less frequent communication as patients became more stable in their sobriety. Providers and clinicians described their process of communication with patients differently, based on whether they worked primarily as a single provider or in a team setting, where they were in connection with social workers, behavioral health providers, client advocates, case management, nursing, and on occasion medical assistants. Diverse patient care settings will necessitate individualization of the data that is presented to the provider. Each patient is in a unique place within their recovery and thus each visit should be patient specific.

*“It kind of depends on the patient. I would think. I think that it'd be really nice to be able to set that and have a number of different choices because you're going to have patients at different stages of addiction and recovery.” -*Participant 4

*“Initially, it's obviously the actual BAC reading, you know, are they abstinent, but by virtue of our philosophy and focus you know, through a period of time, are the data points their awareness of stress […]”.* – Participant 7

When asked about the data they would prefer to see and which data they felt could potentially identify that the patient was at risk for alcohol use, four themes emerged: *Indicators, Visualizations, Prediction* (of BAC or relapse)*,* and *Disengagement* (from the application)*.* The primary high-risk indicators that were requested were breath alcohol content (BAC), mood, and the ability to manage stressors. Visualizations such as trends and patterns, graphs, and color coding were highly recommended as an easy view of who may be at risk for relapse. Integration into the EHR was preferred over accessing the information via a website and quick, easy access was referenced frequently. Providers overwhelmingly preferred that reports and notifications be sent to the EHR and not email. Providers wanted to be able to customize options and reports, with more frequent reports at the beginning of treatment, and the ability to customize based on each patient’s personalized high-risk indicator(s) (e.g., craving level).

*“If there was summary visualizations of this is how the last month has gone that would give me clinically relevant information that I could have done it in a clinical visit. So I think that would probably be most useful.”* - Participant 6

*“I kind of wonder just because the riskier someone's behavior is, the less likely they are to use the device.”* - Participant 2

*“Usually, for me, the EHR would be better because the times that I want to see this dashboard are the times that I'm probably in the EHR.”* - Participant 2

*Accountability* and *Objective Assessment* arose as themes during discussion of why people would use the mobile system. Feedback suggested that having patients self-report on a structured schedule would provide a level of accountability throughout the patient’s recovery. They explained that they expected accountability to be motivating and that this in combination with the frequent, light touchpoints the system had would increase engagement from the patients. Providers also reported using anecdotal evidence from their patients to track progress, as opposed to using objective or objectively verified evidence. Every provider interviewed expressed interest in having an objective measure of alcohol use paired with the ability to identify connections between measures of mood, behavior, or location. Many expressed that this system provided accountability and objective assessment in a better manner than other options they were aware of.

*“Because patient report is just one tool and it's, you know, it's corruptible by whatever the person's motives are in the moment. And so it would be nice to have another piece of data. I think for a patient that's motivated and stable or at least motivated in the moment they start like this device might actually really give them some accountability that might be helpful.” -* Participant 3

In discussing obstacles to use of the mobile system, two themes were identified as provider obstacles*, Information Overload* and *Clinical Relevance*, and two as patient obstacles, *Sustained Engagement* and *Security Concerns*. Providers expressed concerns that the wealth of data that was collected by the application could be overwhelming to providers if it was not conveyed in a concise manner, particularly providers who only discussed AUD progress at 15 min visits. They also suggested that there would need to be an understanding on how to implement the data into the treatment plan, in order to provide clinical relevance.

*“ I would not use it if it was a lot of a lot of data around a small number of patients if I was full time really busy and you know it's like one or two patients that I'm getting seven alerts a day. That one or two patients that would drive me nuts” -* Participant 5

Concerns with *Sustained Engagement* surrounded motivation and compliance with providing breath samples and answering EMA questions, as well as the physical ability of the patients to use the application. Although it was difficult for providers to suggest an amount of non-response time that would signify heightened risk, suggestions of 24 and 72 h indicators were made. The secondary theme that developed in the area of patient obstacles was *Security Concerns*, as there may be apprehension related to the use of facial recognition and geotracking. When asked about potential obstacles to patient engagement, the primary theme that arose was that sustaining patient engagement could be a challenge that would impact successful use of the application. Concerns surrounded motivation and compliance with providing breath samples and answering EMA questions as well as the physical ability of the patients to use the application. They explained that the application may be confusing or complex to patients, that patients may be homeless and not have access to WiFi, electricity for charging, or an address to send items to that they have ordered using the reward of an Amazon gift card, and even that tangible rewards may be more motivating than a gift card. There were also concerns that the devices would be lost, stolen or broken.

*“Facial recognition software has some racist tendencies. They don’t identify people of color as accurately as they identify white people and that makes me nervous. The geolocation also makes me a little bit anxious because I don't know what else that information could be used for.” -* Participant 1

Providers were specifically asked for suggestions for improvement in the functionality, options, reports, and appearance of the system. Two themes emerged; *Increasing Accessibility* and *Bi-directional Communication*. Providers suggested increasing font size, having a read aloud option for people who are hearing impaired and providing multiple language options. Providers also suggested having an ‘Alert’ button where the patient could alert a friend, sponsor or provider when they were struggling.

*“Some of my patients don’t speak English and some of our patients can’t read so there are those pieces that I don’t know if there are any option of making them a bit more inclusive for individuals.” -* Participant 1

## Discussion

CM is among the most effective interventions for substance use disorders, however it has not been widely applied in AUD treatment, in-part because of previous difficulties in detecting alcohol abstinence using standard breath alcohol test procedures [24, 25]. Provider participants in this study believed that ARMS could be useful in the treatment of AUD. They emphasized that recovery was an individualized process and were optimistic that this technology could supplement that patient specific journey. Providers suggested focusing on the following features in the next phases of developing the system: *Indicators, Visualization, Predictors, and Disengagement.* Conversations were awash with descriptions of limited time and the necessity for a quick overview that they could look at, reference, or even use as a discussion tool with patients during a visit. Providers were very interested in using the system for prediction, so they might be able to predict who was at risk for a non-zero or higher BAC. Finally, several providers explained that they felt the best risk indicator would be disengagement from the system.

Providers believed that the system would be a useful addition to treatment by providing *Accountability* for the patient and a method of *Objective Assessment* for the provider. Participants suggested that it will be important to consider *Information Overload* and *Clinical Relevance* when making the next changes to the provider facing system component and to consider *Sustained Engagement* and *Security Concerns* that patients using the system might have. Some of the concerns expressed by the providers also cued the team in to the importance of careful communication about the system. One particular concern, that facial recognition may not work equally well across races, will be important to communicate about clearly in future research and implementation phases with ARMS. While the concern is not relevant to this particular system, as it does not use public databases for facial recognition, but rather uses individually created profiles where faces are matched using an object (face picture of the participant) comparison, people must understand this to negate the concern. Similarly, communication regarding privacy of information such as geolocation must be explicit to help curb security concerns.

Providers also suggested that future modifications would benefit from increased accessibility and methods of communication for patients. Providers had several suggestions on how the system could be modified in the future to increase the breadth of people that could effectively use it. The ability of the system to provide a method of bi-directional communication for patients who find themselves struggling was suggested, such as having an ‘Alert’ button where the patient could alert a friend, sponsor or provider that they are struggling in an effort to engage someone for support before they relapse.

Based on this feedback, system developers and the research team have discussed implementing simple, concise graphs and tables that can be reviewed at a glance with summarized information on patient BAC and EMA responses, as well as indicators of patient engagement with the system. We have now also discussed manners of identifying predictive indicators on an individual patient basis and other ways the system might be able to provide opportunities for providers to individualize it on a patient to patient basis. Practical modifications will also be considered, such as the suggested font size adjustment option, read aloud option and multiple language options. Finally, we will consider a patient facing ‘Alert’ button connected to a friend, sponsor or provider that could be used at the patient’s discretion.

### Limitations

This study has limitations that should be acknowledged. First, while providers came from widely diverse patient care settings, it is important to note that our sample was small (n = 7) and geographically homogeneous (all participants practiced within Idaho and Washington State in the United States), which may have diminished our ability to obtain feedback from a broader number of providers with different treatment experiences. Second, while participants were given a detailed description of how the system functioned, they never interacted with the system itself, which may have restricted their ability to fully understand system functionality. Future iterations of focus group data collection should include actual interaction with the system in order to better estimate and understand its capabilities.

## Conclusion

This study will provide baseline capability for implementation of a remotely monitored CM platform. This technology could be used to supplement tailored treatment and expand access to care. This technology could provide rural populations the ability to connect with care and receive regular feedback from their care team. Information will be used to modify ARMS to make it more user friendly, time saving, secure and relevant to treatment.

## Future directions

Several next steps for this line of research have been identified. A second focus group will be conducted, wherein providers will be able to access and use the ARMS system, to garner feedback on the changes made following this first round of provider feedback. This will allow providers to give additional feedback regarding modifications and the potential utilization of the updated version of ARMS. In addition, we are in the process of finishing a usability study that was planned to recruit n = 20 in order to assess participants’ experience and usability of the software. If successful, larger studies (n > 100) will be sought in order to more definitively determine the efficacy of this type of technology-based CM for alcohol use among rural-dwelling participants.

Within the currently ongoing phase one trial of ARMS, patients have the opportunity for sustained engagement with the technology over the course of 8 weeks. Following their engagement, we solicit patient feedback on usability. We will modify the patient facing system after phase one is completed and solicit patient feedback on visuals provided during phase two of the trial. The provider and patient feedback will provide insight into enhancements that can improve implementation, dissemination and effectiveness.

## Data Availability

The dataset generated and/or analyzed during the current study are not yet available and is in development.

## Abbreviations

CM: Contingency management
AUD: Alcohol use disorders
ARMS: Automated Reinforcement Management System
IOS: Internet operating system
CLS: Crystal L. Smith
NMR: Nicole M. Robin
BAC: Breath alcohol content
EHR: Electronic health record
EMA: Ecological momentary assessment
